# An Audification and Visualization System (AVS) of an Autonomous Vehicle for Blind and Deaf People Based on Deep Learning

**DOI:** 10.3390/s19225035

**Published:** 2019-11-18

**Authors:** Surak Son, YiNa Jeong, Byungkwan Lee

**Affiliations:** Department of Computer Engineering, Catholic Kwandong University, Gangneung 25601, Korea; sonsur@naver.com (S.S.); lupinus07@nate.com (Y.J.)

**Keywords:** autonomous vehicle, audification, sensor, visualization, speech to text, text to speech

## Abstract

When blind and deaf people are passengers in fully autonomous vehicles, an intuitive and accurate visualization screen should be provided for the deaf, and an audification system with speech-to-text (STT) and text-to-speech (TTS) functions should be provided for the blind. However, these systems cannot know the fault self-diagnosis information and the instrument cluster information that indicates the current state of the vehicle when driving. This paper proposes an audification and visualization system (AVS) of an autonomous vehicle for blind and deaf people based on deep learning to solve this problem. The AVS consists of three modules. The data collection and management module (DCMM) stores and manages the data collected from the vehicle. The audification conversion module (ACM) has a speech-to-text submodule (STS) that recognizes a user’s speech and converts it to text data, and a text-to-wave submodule (TWS) that converts text data to voice. The data visualization module (DVM) visualizes the collected sensor data, fault self-diagnosis data, etc., and places the visualized data according to the size of the vehicle’s display. The experiment shows that the time taken to adjust visualization graphic components in on-board diagnostics (OBD) was approximately 2.5 times faster than the time taken in a cloud server. In addition, the overall computational time of the AVS system was approximately 2 ms faster than the existing instrument cluster. Therefore, because the AVS proposed in this paper can enable blind and deaf people to select only what they want to hear and see, it reduces the overload of transmission and greatly increases the safety of the vehicle. If the AVS is introduced in a real vehicle, it can prevent accidents for disabled and other passengers in advance.

## 1. Introduction

Autonomous cars represent a key area of the fourth industrial revolution. Various carmakers around the world are actively conducting research with the aim of producing fully autonomous vehicles, and advances in information and communications technology (ICT) are greatly speeding up the development of autonomous vehicle technology. A fully autonomous vehicle means that people are not involved in driving at all, the car drives on its own and immediately deals with a variety of risk factors. Autonomous vehicles can be classified into five stages. Level 0 is the stage where the driver performs all actions to drive the vehicle, and there is no autonomous driving at all. Level 1 is the passive stage, where the vehicle automatically handles acceleration, steering, etc. In addition, vehicles in this stage have a lane-keeping assist (LKA), which automatically returns the vehicle to the original lane when it gets out of the lane without turning on a turn signal, and cruise control (CC), which maintains a specified speed. Level 2 is the stage where the vehicle automatically decelerates and even operates the brakes, which is slightly more advanced than Level 1.

Level 3 is a semi-autonomous driving stage, which includes all the functions of Level 2 and analyzes the road situation using advanced sensors or radar so that the car can drive a certain distance on its own without driver intervention. Level 4 is the stage where a self-driving vehicle can safely reach the designated destination without the driver’s intervention. However, this is not a perfect stage because it cannot completely guarantee safety. At Level 5, the driver does not exist, there are only passengers, and the vehicle performs all movements on its own. The vehicle uses artificial intelligence and various sensors to cope with all possible road situations [1]. 

Many companies, including Google^®^, Tesla, and Mercedes-Benz, are testing fully autonomous driving vehicles on the road. Google’s self-driving car, however, collided with a large bus, while Tesla’s autonomous driving vehicle crashed into a bicycle and caused a fire [2,3]. Fully autonomous driving vehicles are not yet available in the test phase. In particular, since occupants of fully autonomous vehicles do not drive directly on their own, it is not easy to recognize the situation before and after a vehicle accident, and also, they are likely to be negligent in checking the vehicle. Therefore, fully autonomous vehicles should frequently inform the occupants of their analysis results through artificial intelligence-based self-diagnosis. If all passengers of a fully autonomous vehicle are deaf or blind, there is no way to inform them of the results of the self-diagnosis analysis, which increases the risk of an accident. In 2016, Google succeeded in piloting a self-driving vehicle with a blind person, but even then, he was in the vehicle with a sighted person [4].

To address these problems, this paper proposes an audification and visualization system (AVS) for blind and deaf people. The AVS consists of a data collection and management module (DCMM), an audification conversion module (ACM), and a data visualization module (DVM). The DCMM stores and manages the data collected from the vehicle. The ACM has a speech-to-text submodule (STS) that recognizes a user’s speech and converts it to text data, and a text-to-wave submodule (TWS) that converts text data to voice. The DVM visualizes the collected sensor data, fault self-diagnosis data, etc., and places visualized data according to the size of the vehicle’s display.

The composition of this paper is as follows: Section 2 describes the existing studies related to the AVS in this paper. Section 3 details the structure and operation of the AVS. Section 4 compares it with the existing methods to analyze performance. Section 5 discusses the conclusion of the proposed AVS and future research directions.

## 2. Related Works 

### 2.1. Hidden Markov Model

Li et al. proposed a new algorithm combining the hidden Markov model (HMM) and Bayesian filtering (BF) techniques to recognize a driver’s intention to change lanes. The grammar recognizer in the algorithm was inspired by speech recognition, and the output value of the algorithm is preliminary classified behavior. The behavior classification value, the final output of BF, is generated using the current and previous output of the HMM. This algorithm was validated using a naturalistic dataset. The proposed HMM–BF framework can meet 93.5% and 90.3% recognition accuracy for right and left lane changes, respectively, which is a significant improvement over the HMM-only algorithm [5].

Liang et al. proposed a new filter model-based hidden Markov model (FM-HMM) for intrusion detection system (IDS) to decrease the overhead and time for detection without impairing accuracy. This work was the first to model the state pattern of each vehicle in vehicle ad hoc networks as an HMM and quickly filter the messages in the vehicle instead of detecting these messages. The FM-HMM consists of three modules: The schedule module generates the parameters of the HMM for adjacent vehicles by using the Baum–Welch algorithm [6]; the filter module predicts the future state of an adjacent vehicle by using several HMMs; and the update module updates the parameters of the HMM using the timeliness method [7].

Saini et al. introduced two additional kernels based on convex hull and the Ramer–Douglas–Peucker (RDP) algorithm and proposed a trajectory classification approach, which supervises a combination of global and segmental HMM-based classifiers. To begin with, the HMM is used for global classification of categories to provide state-by-state distribution of trajectory segments. The trajectory and global recognition that completed classification improved the classification results. Finally, global and segmental HMM are combined using a generic algorithm. They experimented with two public datasets, commonly known as T15 and MIT, and achieved accuracy of 94.80% and 96.75%, respectively, on these datasets [8].

Siddique et al. presented a self-adaptive sampling (SAS) method for mobile sensing data collection. SAS regulates the sampling rate using the flow state of vehicles estimated in individual lanes, classifying the estimated flow state into four categories (free flow, stopped, acceleration, and deceleration) using the HMM and identifies stopping and movement in the lane using support vector machine (SVM). The identification of vehicle flow conditions is used to change the sampling rate. The SAS method can reduce the total amount of data by 67–77% while retaining the most important data points [9].

Liu et al. proposed a controller integration approach that adopts behavior classification to improve the ability of leading vehicles to cope with outside obstacles. This approach, based on the HMM, detects if there is any hazard behavior in the neighboring vehicles. The detected behaviors are transmitted to the model predictive controller in a driving vehicle. A behavior-guarded cost function of the controller is designed to increase the stability against danger while driving. The effect of the state deviation of the lead vehicle in the convoy is studied based on leader-to-formation stability characteristics. Furthermore, a nonlinear bound is also given to specify the performance of the proposed controller [10].

Mingote et al. proposed a novel differentiated neural network with an alignment mechanism for text-dependent speaker verification. They did not extract the embedding of speech from the global average pooling of the temporal dimension. Because the proposed neural network uses phonetic information for verification, it maintains the temporal structure of each phrase by replacing a redirection mechanism with the alignment model of a phonetic phrase. They applied convolutional neural networks (CNNs) to the front end and learn the neural network that produces super-vectors of each word, whose pronunciation and syntax are distinguished at the same time. This choice has the advantage that super-vectors encode phrases and speaker information, which showed good performance in text-dependent speaker verification tasks [11].

Wang et al. proposed a method for determining vehicle driving status from time-ordered trajectory data using the HMM. This method is preprocessed to discard track sequences with insufficient length to ensure the usefulness of linear smoothing and least squares fitting. A directional area segmentation algorithm was proposed to extract the directional angle of the vehicle from the preprocessed orbital sequences, and it obtains and patterns the various driving states of the vehicle in real time. Finally, multiple observations based on the Baum–Welch algorithm can obtain the optimal HMM model parameters for each track pattern at a particular traffic site and then determine the real-time vehicle driving state by matching with the trained HMM model above [12].

Kato et al. proposed a car tracker based on a hidden HMM/Markov random field (MRF)-based segmentation method that is capable of classifying each small region of an image into three categories (vehicles, shadows of vehicles, and background) from a traffic-monitoring video. The temporal continuity of the different categories for one small location is modeled as a single HMM along the time axis, independent of the neighboring regions. In order to incorporate spatially dependent information among neighboring regions into the tracking process, at the state-estimation stage, the output from the HMMs is regarded as an MRF and the maximum a posteriori criterion is employed in conjunction with the MRF for optimization. At each time step, the state estimation for the image is equivalent to the optimal configuration of the MRF generated through a stochastic relaxation process [13].

Wang et al. proposed a novel framework called chain of road traffic incident (CRTI) for predicting accidents. CRTI observes the moving features of a driving vehicle, which are the external performance of a road transport system that reflects the “health states” (safety states) of a given time. A two-stage modeling procedure for CRTI is then proposed using a scenario-based strategy. A support vector machine is used to classify leaving versus remaining in lane scenes, and Gaussian mixture-based hidden Markov models are developed to recognize accident versus non-accident pattern CRTIs given the classified scene [14]. 

Jazayeri et al. presented a comprehensive approach to localize target vehicles with video under various environmental conditions. Extracted geometric features from the video are continuously projected onto a 1-D profile and constantly tracked. This method compensates for the complexity of the vehicle shape, color, and type recognition using time information and models the driver’s field of vision probabilistically according to the features of the background and the movement of the vehicle. In the proposed method, the HMM is used to probabilistically track vehicles apart from the background [15].

### 2.2. Vehicle for Disabilities

Choromański et al. presented an original concept of an urban transport system based on a hybrid vehicle that can move by a human-driven electric vehicle or a special pod car vehicle (right of way a, b, or c). The system was developed at the Warsaw University of Technology and is referred to as the hybrid vehicle and transit system for urban application (HVTASUA). The system was designed not only for ordinary drivers, but also for elderly people and those who lack driving skills, such as people with physical disabilities. Based on this system, an original design for vehicles and standardization of human-machine interface (HMI) are proposed. This HVTSUA is integrated with Ford’s already developed Eco-Car system. Integrating these two elements and equipping them with new technology became the basis for a system with new quality [16].

The aim of Bennett et al. was to investigate possible barriers to the use of autonomous vehicles (AVs) that are perceived by people with intellectual disabilities. A structural topic modelling (STM) approach was employed to analyze 177 responses of mentally disabled people to an open-ended question about AV travel intentions. Results from the STM, together with data on the sample participants’ level of internal locus of control, generalized anxiety, age, gender, prior knowledge of AVs, and level of individual disability were then incorporated into a structural equation model constructed to relate attitudinal topics identified by the STM to the participants’ willingness to travel in AVs. Three categories of attitudes toward AVs arose from the STM, relating to freedom, fear, and curiosity. Two of the three themes, freedom and fear significantly predicted the participants’ willingness to use driverless vehicles. The freedom theme was significantly explained by generalized anxiety, intensity of disability, and prior knowledge of AVs. The concept of fear depended significantly on generalized anxiety and prior knowledge, and also on the locus of control and (female) gender. The theme of curiosity was influenced by locus of control and prior knowledge [17]. They employed a mixed research methodology to assess attitudes toward AVs in a UK sample of individuals with physical disabilities affecting their mobility. Participants were asked in an open-ended way to express their ideas about AVs, and their responses were analyzed using STM. Outputs for the STM analysis were then adopted in a structural equation model (SEM) constructed to predict the willingness of the participants to travel in driverless vehicles. The results were compared with those obtained from a control group of people without physical disabilities. The attitudes of people with disabilities toward AVs were significantly different from those of respondents without disabilities. Attitudes toward AVs among people with disabilities were significantly influenced by their level of interest in new technologies, generalized anxiety, intensity of disability, prior knowledge of AVs, locus of control, and action orientation. A latent class analysis confirmed the relevance of these variables as determinants of the views of people with disabilities on AVs [18].

Xu et al. proposed an intelligent guided vehicle prototype for blind people. The system integrates ultrasound and photoelectric detection using ARM as a controller and processor, and automatically navigates to the destination. This system consists of four modules: ultrasonic detection, photoelectric detection, voice prompt, and automatic control. The ultrasonic detection module detects reflected signals on the road using the distance between the road block and the vehicle. The photoelectric detection module recognizes the road and tracks the vehicle. The voice prompt module and automatic control module allow blind people to enter voice commands and control the vehicle [19].

## 3. Proposed Method

### 3.1. Overview

An autonomous driving vehicle performs a number of driving functions using various sensors. Although the sensor data measured in the vehicle varies, there is a limit to the type of data displayed on the instrument cluster and the way it is indicated. For example, the vehicle’s instrument cluster displays some information about the vehicle, such as speed, RPM, etc., but much other information, such as air pressure, door opening and closing, etc., is still not accurately displayed. If all of this information were to be displayed on the instrument cluster, it would be difficult for occupants to look at so much information at a glance. It goes without saying that if only blind or deaf people are riding in self-driving cars, it would be more difficult to identify the information. The status information of the vehicle should be visualized more accurately for the deaf and be audified for the blind.

**Figure 1:**
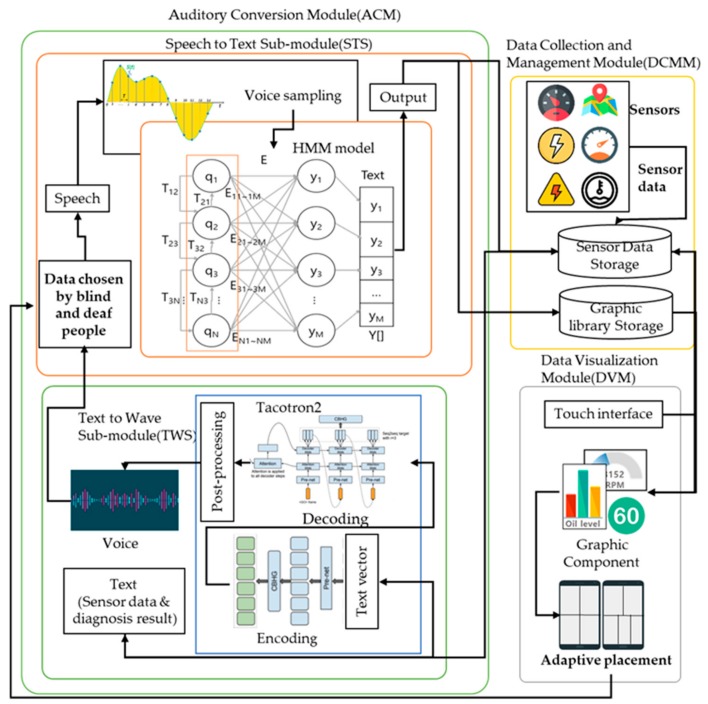
Structure of the system. HMM: hidden Markov model.

To solve these problems, this paper proposes an audification and visualization system (AVS) of an autonomous vehicle for blind and deaf people based on deep learning, which uses self-diagnosis results published previously [20,21] and the sensor data collected from vehicles, a graphical library to visualize the data desired by deaf people and audify the data desired by blind people. Figure 1 shows the structure of the AVS, which consists of three modules. 

The data collection and management module (DCMM) stores and manages all the sensor data collected from the vehicle, self-diagnosis data, and graphics libraries in in-vehicle storage. The auditory conversion module (ACM) receives speech and provides voice output about the vehicle’s condition. The ACM consists of a speech-to-text submodule (STS) that recognizes speech and converts it to text data and a text-to-wave submodule (TWS) that converts data to voice. The STS learns HMM point-to-point using the in-vehicle NVIDIA px2 driver and receives a sensor name from the user by using the learned HMM. The TWS converts text data to voice using Tacotron2 and outputs it. The data visualization module (DVM) visualizes the data received from the DCMM and places the visualized data, the graphic components, on an in-vehicle display.

The AVS operates as follows. First, the AVS receives information visualized through the touch interface or audified through a speech recognizer. If someone uses speech recognition, the ACM converts the speech to text using the HMM of the STS and sends the converted text to the CMM. The CMM transmits the vehicle’s sensor data and self-diagnosis data [20,21] to the ACM, and the ACM audifies the information received from the DCMM.

If someone uses the touch interface, the DCMM selects the sensor data in the sensor data storage and the graphics functions in the graphic library storage and transmits them to the DVM. The DVM visualizes the data entered by the DCMM and the vehicle’s self-diagnostic data and positions the visualized data on the vehicle’s display adaptively to inform the person of the vehicle’s condition.

### 3.2. Design of a Data Collection and Management Module

**Figure 2:**
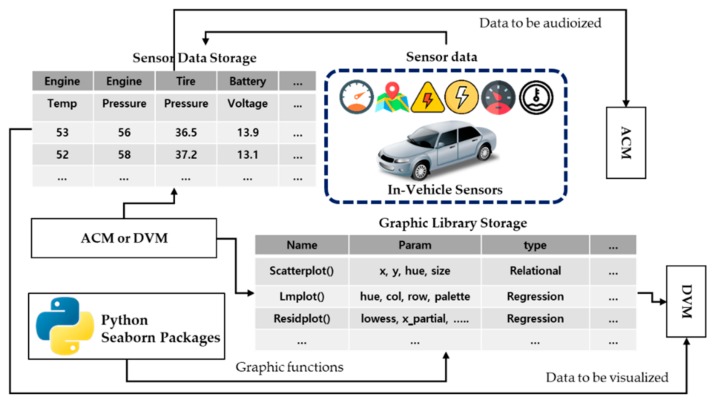
Structure of a data collection and management module.

The DCMM stores the sensor data and Python seaborn packages collected from the vehicle. It consists of a transceiver that receives in-vehicle sensor messages, sensor data storage where the received messages are stored, and graphic library storage where the graphic libraries are stored. The transceiver consists of a CAN transceiver that receives CAN messages, a MOST transceiver that receives MOST messages, a FlexRay transceiver that receives FlexRay messages, and a LIN transceiver that receives LIN messages. Each received message is stored in the sensor data storage, as shown in Figure 2. 

When the ACM or DVM requests the audified or visualized data to the DCMM, the DCMM retrieves them from sensor data storage and transmits them to the ACM or DVM again and also provides the functions necessary for the DVM to visualize the data in graphic library storage. 

The DCMM then stores the condition of the vehicle using the previously published self-diagnosis system and transmits it to the ACM and DVM, so that the self-diagnosis results of the autonomous vehicle are visualized or audified.

### 3.3. Design of an Auditory Conversion Module

**Figure 3:**
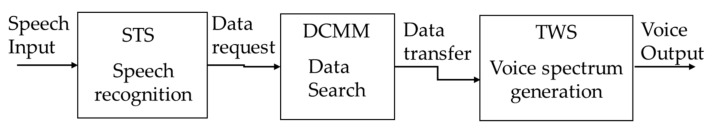
Structure and data flow of ACM.

The auditory conversion module (ACM) receives the user’s speech and provides voice output of the vehicle’s condition. Figure 3 shows the flow of the ACM. The ACM consists of a speech-to-text submodule (STS) that recognizes speech and converts it into text data and a text-to-wave submodule (TWS) that converts text data to voice. The ACM receives the data that has to be audified by using the STS. The ACM transmits the received data to the DCMM, and the DCMM again transmits the data that has to be audified to the ACM. The ACM then generates sentences with the data, which have to be audified using the TWS and delivers them after changing to voice. For example, if a person says, “Tell me the brake status when the sound is strange,” the STS recognizes it and requests the information about the brake sensors and the vehicle’s self-diagnosis information to the DCMM. The DCMM transmits the brake information and the vehicle’s self-diagnosis information to the TWS, and the TWS informs the user after turning them into voice.

#### 3.3.1. Speech-to-Text Submodule (STS) 

**Figure 4:**
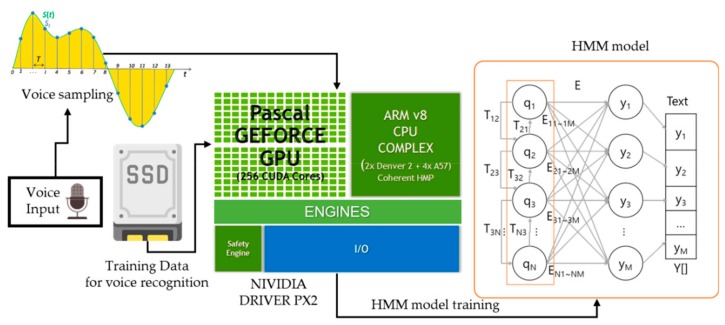
Point-to-point learning order of the hidden Markov model (HMM).

The STS uses the HMM to recognize speech. Unlike a recurrent neural networks (RNN), which reflects all previous states, the HMM recognizes the user’s voice based on the Markov chain, which relies on the immediately previous state. Because the STS recognizes in a short word the data that have to be visualized in a short word, the HMM is more efficient than the RNN. The STS learns the HMM independent of the cloud and receives speech from the vehicle’s microphone and outputs the text data by using a point-to-point learning method and NVIDIA PX2 driver. An in-vehicle solid-state drive (SSD) transmits training data to the NVIDIA PX2. The NVIDIA PX2 learns the HMM using it. The learned HMM converts speech to text data. The ACM transmits the converted text data to the DCMM. Figure 4 shows the learning order of the HMM.

(1) $$P\left(q_{i}|q_{1},\;\ldots ,\;q_{i-1}\right)=P(q_{i}|q_{i-1})\;.$$

**Figure 5:**
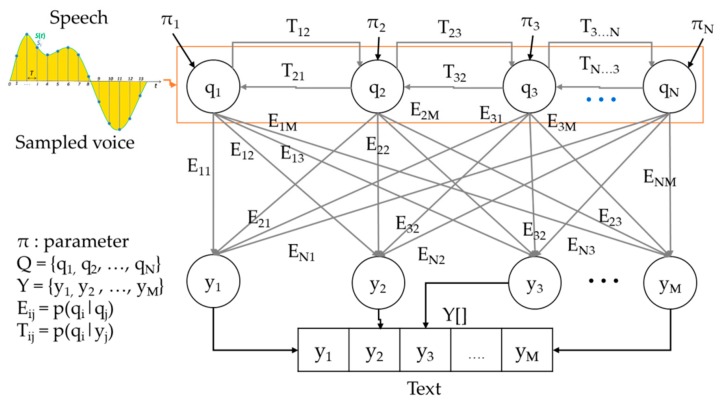
Flowchart of HMM.

Equation (1) represents a Markov chain. The Markov chain can compute the results of perfect input and output. However, the HMM, which has to compute the results with only a person’s input, uses the Markov chain assuming that the results are hidden. In general, the HMM is represented in the form of a directed graph, as shown in Figure 5. In the graph, q_i_ represents a hidden state and y_j_ refers to the observed value from q_i_. The HMM consists of <Q, Y, π, T, E>, and Table 1 shows the components of the HMM.

When *θ* is given in the HMM, the STS computes a probability about a given observed value by using a dynamic programming-based forward algorithm and a list of the states with the highest probability by using the Viterbi algorithm. Viterbi Algorithm is a dynamic programming technique for finding the most likely sequence of hidden states. Here, the hidden states refer to observed value of the HMM.

When the list of states is computed, the STS learns the HMM’s *θ* by using the Baum–Welch algorithm and the training data. In other words, the STS computes the probability of observed values and the list of states by specifying the initial *θ* and learns the HMM based on them. Because the STS outputs part names by receiving the user’s speech and the observed STS value, *Y* becomes the part name of a vehicle that can be audified, and these are shown in Table 2.

To begin with, the STS sets a random initial θ and computes the observed value *P(Y|θ)*. Equation (2) computes the first observed value, y_1_, and Equation (3) computes y_1_–y_m_:

(2) $$P\left(y_{1}|\theta \right)=\sum_{i\;1}p\left(q_{i\;1}\right)p(y_{1}|q_{i\;1}),$$

(3) $$P\left(Y|\theta \right)=\;\underset{i\;1}{\sum }\underset{i\;2}{\sum }\ldots \underset{i\;m}{\sum }p\left(q_{i\;1}\right)p(y_{1}|q_{i\;1})p(q_{i\;2}|q_{i\;1})p(y_{2}|q_{i\;2})\ldots p(q_{i\;m}|q_{i\;m-1})p(y_{m}|q_{im}).$$

However, because this method has the time complexity of $O\left(n^{m}\right),$ it is impossible to compute the observed values inside the vehicle. Therefore, this paper uses the forward algorithm in the HMM to reduce the computation time of the STS. The key idea of the forward algorithm is to store duplicate computation results in a cache and fetch them when necessary. The forward algorithm defines the new variable $\alpha_{t}\left(p_{j}\right)$ in Equation (4). Using the defined $\alpha_{t}\left(p_{j}\right)$, Equation (3) is simplified to Equation (5):

(4) $$\alpha_{t}\left(q_{j}\right)=p\left(y_{1},y_{2},\ldots ,\;y_{t},\;s_{t}=q_{j}|\theta \right),$$

(5) $$\alpha_{t}\left(q_{j}\right)=\overset{N}{\underset{i=1}{\sum }}a_{t-1}\left(q_{i}\right)p\left(q_{j}|q_{i}\right)p\left(y_{t}|q_{j}\right).$$

When the observed values are computed, the STS traces back the hidden states with the observed values and makes the back-traced states into one array by using the Viterbi algorithm. Algorithm 1 represents the pseudo-code in which the STS computes observed values using the forward algorithm and an array of hidden states by using the Viterbi algorithm.
**Algorithm 1.** Computation of observed values and array of states.
Input: initial probabilities π, transition probabilities T,
    emission probabilities E, number of states N,
    observation Y = $y_{1},\;y_{2},\;\ldots ,\;y_{m}$; Forward(π, T, E, Y){ for(j=1; jN; j++){  $\alpha_{1}\left(q_{j}\right)=p\left(q_{j}\right)p(y_{1}|q_{j})$ } for(t=2; t<=T, t++){  for(j=1; j<=N; j++){
    $\alpha_{t}\left(q_{j}\right)=\sum_{i=1}^{N}a_{t-1}p\left(q_{i}\right)p(q_{j}|q_{i})p(y_{t}|q_{j})$  } } p(Y|π, T, E) = $\sum_{i=1}^{N}a_{T}\left(q_{j}\right)$ return p(Y|π, T, E) } Viterbi(π, T, E, Y){ for(j=1; j<=N; j++){  $v_{1}\left(q_{j}\right)=p\left(q_{j}\right)p(y_{1}|q_{j})$ } for(t = 2; t<=T, t++){  for(j=1; j<=N; j++){    $v_{t}\left(q_{j}\right)=\underset{q\in Q}{\max}v_{t-1}p\left(q\right)p(q_{j}|q)p(y_{t}|q_{j})$    S[t] = $\underset{q\in Q}{\arg\;\max}v_{t-1}p\left(q\right)p(q_{j}|q)p(y_{t}|q_{j})$  } } return S[] }

The STS learns the HMM by using the computed observed values, an array of states, and the Baum–Welch algorithm. The HMM’s learning is to make the best parameter, θ*. The Baum–Welch algorithm computes a parameter *θ* using correct *Y* and *Q* in Equation (6):

(6) $$P\left(Y,\;Q|\theta \right)=\;\overset{N}{\underset{k=1}{\prod }}\left(p\left(q_{1}^{k}\right)p\left(y_{1}^{k}|q_{1}^{k}\right)\overset{M}{\underset{t=2}{\prod }}(p\left(q_{t}^{k}|q_{t-1}^{k}\right)p\left(y_{t}^{k}|q_{t}^{k}\right)\right).$$

However, because the STS does not know the exact *Q* for *Y*, the STS converts Equation (6) to Equation (7) by taking the log on both sides of Equation (6) and computes $Q\left(\theta ,\theta^{\prime }\right)$ by substituting Equation (7) with Equation (8):

(7) $$\log p(Q,\;Y|\theta )=\overset{N}{\underset{k=1}{\sum }}\{p\left(q_{1}^{k}\right)p\left(y_{1}^{k}|q_{1}^{k}\right)+\overset{M}{\underset{t=2}{\sum }}\log(p\left(q_{t}^{k}|q_{t-1}^{k}\right))+\overset{M}{\underset{t=2}{\sum }}\log\left(p\left(y_{t}^{k}|q_{t}^{k}\right)\right)\}p\left(Q,Y|\theta^{\prime }\right),$$

(8) $$Q\left(\theta ,\theta^{\prime }\right)=\underset{q_{1},\;q_{2},\;\ldots ,\;q_{N}}{\sum }\log\left\{p\left(Q,Y|\theta \right)\right\}p\left(Q,Y|\theta^{\prime }\right).$$

In Equation (7), θ means a current parameter and $\theta^{\prime }$ is the immediately previous one. The STS substitutes Equation (7) with Equation (8) and generates $L\left(\theta ,\theta^{\prime }\right)$ that can compute $Q\left(\theta ,\theta^{\prime }\right)$ using the Lagrange multiplier method [22]. Equation (9) indicates $L\left(\theta ,\theta^{\prime }\right)$:

(9) $$L\left(\theta ,\theta^{\prime }\right)=Q\left(\theta ,\theta^{\prime }\right)-\omega_{\pi }\left(\overset{N}{\underset{i=1}{\sum }}p\left(q_{i}\right)-1\right)-\;\overset{N}{\underset{i=1}{\sum }}\left(\overset{N}{\underset{j=1}{\sum }}p\left(q_{j}|q_{i}\right)-1\right)-\overset{N}{\underset{i=1}{\sum }}\omega_{E_{i}}\omega_{T_{i}}\left(\overset{M}{\underset{j=1}{\sum }}p\left(y_{j}|q_{i}\right)-1\right).$$

In Equation (9), $p\left(q_{i}\right)$ means π_i_ of θ, $p\left(q_{j}|q_{i}\right),$ Tij of θ, $p\left(y_{j}|q_{i}\right)$, Eij of θ, and ω, Lagrange multiplier. Because the STS has to find θ that maximizes $L\left(\theta ,\theta^{\prime }\right)$, it computes the optimal parameter $\theta^{*}$ by differentiating $L\left(\theta ,\theta^{\prime }\right)$ with π_i_, T_ij_, and E_ij_. Equation (10) computes the optimal π_i_ value, Equation (11) the optimal T_ij_ value, and Equation (12) the optimal E_ij_ value:

(10) $$\pi_{i}=\frac{\sum_{k=1}^{K}p(Y,\;q_{1}^{k}=\;q_{i}|\theta^{\prime })}{\sum_{j=1}^{N}\sum_{k=1}^{K}p(Y,\;q_{1}^{k}=\;q_{i}|\theta^{\prime })},$$

(11) $$T_{\mathrm{ij}}=\frac{\sum_{k=1}^{K}\sum_{t=2}^{T}p(s_{t-1}^{k}=q_{j},s_{t}^{k}=q_{j}{|Y}^{k},\;\theta^{\prime })}{\sum_{k=1}^{K}\sum_{t=2}^{T}p(s_{t}^{k}=q_{j}{|Y}^{k},\;\theta^{\prime })}\;,$$

(12) $$E_{\mathrm{ij}}=\;\frac{\sum_{k=1}^{K}\sum_{t=1}^{T}p(s_{t}^{k}=q_{i}{|Y}^{k},\;\theta^{\prime })I\left(y_{t}^{k}=q_{j}\right)}{\sum_{k=1}^{K}\sum_{t=1}^{T}p(s_{t}^{k}=q_{i}{|Y}^{k},\;\theta^{\prime })}.$$

If Equations (10)–(12) are used, it is possible to compute the optimal parameter $\theta^{*}$ from the previous parameter θ. When the STS computes the optimal parameter $\theta^{*}$ using the initial parameter, the STS receives speech and transmits a list of text data for audification to the DCMM.

#### 3.3.2. Text-to-Wave Submodule

**Figure 6:**
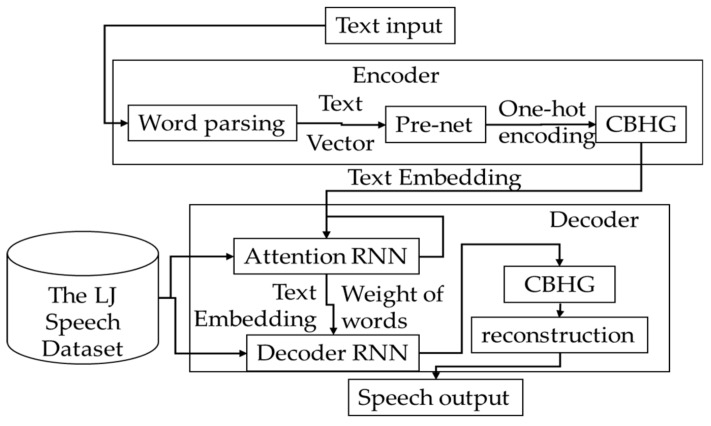
Structure of TWS.

The text-to-wave submodule (TWS) audifies the sensor data transmitted from the DCMM. It executes text-to-speech (TTS) using Google’s Tacotron2 [23]. Because the Tacotron2 operates on the basis of an RNN encoder–decoder model, in this paper Tacotron2 is learned by using the LJ Speech Dataset [24] and estimates whether the sensor data of the vehicle are accurately output to voice. Figure 6 shows the flow of the TTS.

To begin with, the TWS receives sentences and breaks the words in the encoder. When a vector of the words is entered in the encoder, the pre-net of the Tacotron2 one-hot encodes the vector. Here, one-hot encoding means the process of converting a text vector into an array of 0 and 1 that the encoder can recognize easily. 

**Figure 7:**
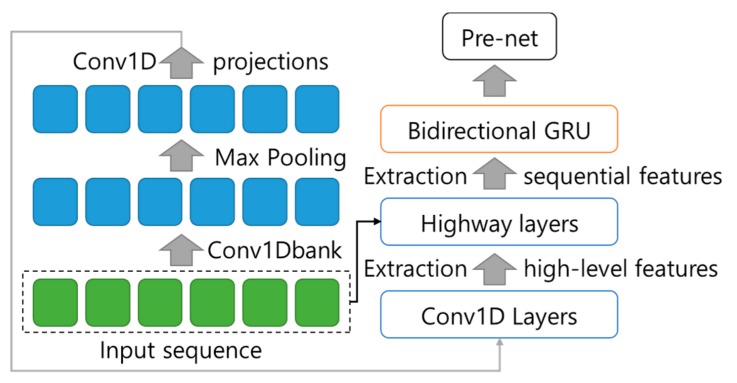
The structure of the convolution bank + highway net + bidirectional gated recurrent unit CBHG.

When the pre-net one-hot encodes the text vector, the TWS transmits it to the Tacotron2’s convolution bank + highway net + bidirectional gated recurrent unit (CBHG) network, a neural network model. The convolution bank extracts the features of text from one-hot-encoded text vectors, the highway net deepens the neural network model, and the bidirectional gated recurrent unit (GRU) generates text embedding by considering the previous and subsequent information of vectors processed in pre-net. The input sequence of the Convolution Bank is K sets of 1-D convolutional filters. Where 1 set of 1-D convolutional filters consists of C_1_, C_2_, C_3_ … and C_K_ filter. The input sequence is max-pooled. The output of the Convolution Bank is delivered to the Highway Net which extracts high-level features from it. Finally, the Bidirectional GRU extracts sequential features using the order of the input sequence. Figure 7 shows the structure of the CBHG.

**Figure 8:**
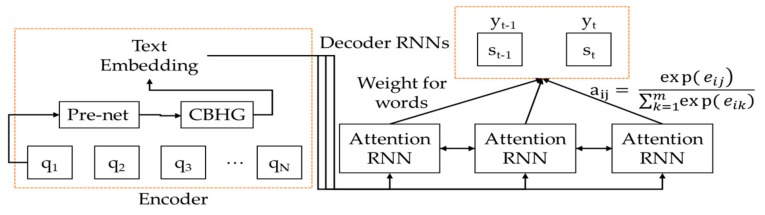
Importance of words computed in attention RNN.

The generated text embedding is sent to an attention model. The attention model determines which is more complex and which is more important in the transmitted text embedding by using the attention RNN. This attention model enables the Tacotron2 to do point-to-point learning and converts to voice the text vectors that are not learned. Figure 8 shows the composition of the attention and decoder RNNs. Equation (13) indicates the attention value used in the attention RNN:

(13) $$\mathrm{Attention}\;\left(Q,\;K,\;V\right)=\;\mathrm{Attention}\;\mathrm{value},$$

where Q means the query of the hidden states that decoder cells have at the time of *t*, K means the keys of the hidden states that encoder cells have at all times, and V means the values of the hidden states that encoder cells have at all times. 

The attention RNN sends the attention values, the weight of each word, to the decoder RNN. Attention value a is obtained from Equation (14):

(14) $$a_{\mathrm{ij}}=\;\frac{\exp\left(e_{ij}\right)}{\sum_{k=1}^{m}\exp\left(e_{ik}\right)}.$$

Here, a_ij_ means the weight of the input states. An input state with high weight has a greater influence on the output state. The a_ij_ is normalized to 1. The m in Equation (14) means the number of words entered in the decoder, and a_ij_ indicates how similar the *i*th vector of text embedding and the *j*th vector of the encoder are in the previous step. The e is computed in Equation (15). In Equation (15), α represents a constant to optimize the similarity between s(i–1) and q_j_ like the learning rate of a neural network model, s(i–1) represents the text embedding vector of the previous step when the decoder predicts the *i*th word, and q_j_ represents the *j*th column vector of the encoder.

(15) $$e=\;\alpha \left(s_{i-1},\;q_{j}\right).$$

The text embedding that the weight computed in the attention RNN is added to is sent to three decoder RNNs. The decoder RNNs convert the text embedding to a spectrum form by using a sequence-to-sequence method. The converted text embedding is output to voice via the CBHG module.

### 3.4. Design of a Data Visualization Module

**Figure 9:**
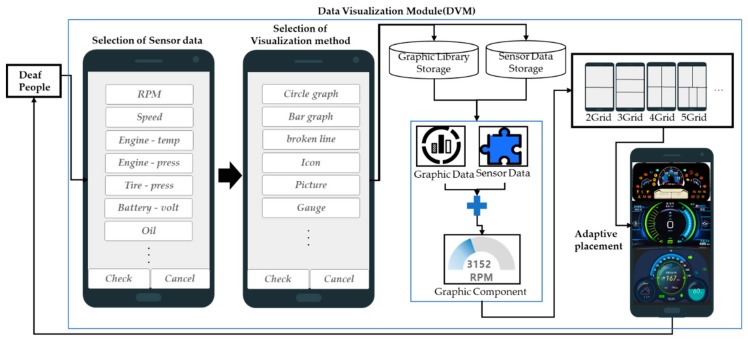
Structure of data visualization module.

The data visualization module (DVM) receives all the sensor data collected from the vehicle and visualizes only the data that deaf people desire by using an adaptive component placement algorithm that adjusts and places the graphical user interface (GUI) components properly on the vehicle’s display. Figure 9 shows the structure of the DVM.

The visualization process of the DVM is as follows. First, people can select the sensor data to visualize. The types of sensor data are represented by buttons. Second, they decide how they want to visualize the sensor data of their own choosing. The sensor data that will be visualized has to be selected before selecting the visualization method. For example, if someone wants to display RPM as a bar graph, they choose RPM from the sensor data and click the bar graph from the visualization method. The DVM generates a GUI component combining the entered sensor data and visualization method. Algorithm 2 indicates the process by which the DVM receives the necessary information for visualization. 

**Algorithm 2.** Data visualization algorithm.
input: int number_kind, list data_value[], String data_name[], String   graph_name; init: Button check_sensors[number_kind];   List graph[];   List param_data[];   int k=0; for(int i = 0; i<kind; i++){ check_sensors[i]=data_value[data_name[i]]; check_sensors.enable; } if(ClickEvent(data_name) && ClickEvent(graph_name)){ check_sensors[data_name].disable; param[k].input(key : data_value[data_name],value : graph[graph_name],); } if(ClickEvent(send)){ send(param[]); } if(ClickEvent(cancel)){ check_sensors[data_name].enable; param[k].delete(key : data_value[data_name],value :  graph[graph_name],); }

Here, *number_kind* means the number of data types output to the display, *data_value[]* means sensor values, *data_name[]* means sensor names, and *Graph_name* is a visualization method. The selected data types are stored in *data_value[data_name]* and the selected visualization method in *value: graph[graph_name]*. *ClickEvent(send)* means that the selection and visualization of sensor data is over, and *ClickEvent(cancel)* means that all selected contents are deleted. When someone finishes all selections, the DVS generates a GUI component by combining the selected sensor name, sensor data, and visualization method.

The DVS then receives and visualizes the vehicle’s failure self-diagnosis from the DCMM. The visualization method of failure self-diagnosis is not selected manually, it is visualized using a gauge graph. The DVS sets the gauge graph to a range from 0 to 1 to visualize the failure self-diagnosis. Because the type of display and the resolution size used by each vehicle vary, it is difficult to adapt to various environments unless the sizes of visualization components vary. Thus, the DVM generates adaptive components that adjust the size of the GUI components and displays them on the vehicle’s display so that visualization components can be used without problems in various display environments. Algorithm 2 shows the process of dividing the display and placing GUI components.

**Algorithm 3.** Adaptive component placement.
SetComponent(Object[] component[], int compNumber, int compX[], int compY[]){ int i = 0; Rect grid[] = GridPartition(compNumber, vertical, 1.5); for(i = 0; i<= compNumber; i++){  if(compNumber == 1)   displayComponent(component[i], grid[i]);  else{   if((compX[i]<compY[i]) && (grid[i].x<grid[i].y)){    displayComponent(component[i], grid[i]);   }   elseif((compX[i]>compY[i]] && (grid[i].y<grid[i].x)){    displayComponent(component[i], grid[i]);   }   elsief((compX[i]<compY[i]] && (grid[i].y<grid[i].x)    && (component[i].type == digit)){    component[i] = ReplaceXandY(component[i]);    displayComponent(component[i], grid[i]);   }   else if((compX[i]<compY[i]) && (grid[i].y<grid[i].x)
    && (component[i].type != digit)){    grid[i] = ReplaceGrid(horizontal, 1.5);    displayComponent(component[i], grid[i]); }}}}

Here, *compNumber* means the number of components, *compX* means the size of the component’s x-axis, and *compY* the size of the y-axis. *GridPartition (compNumber, Vertical, 1.5)* means that when a grid is divided, it is divided vertically in proportion to the number of components. In *i = 0*; *i < compNumber; i++*, *i* is the number of components in the grid, and grid segmentation is done as large as the number of components.

If the order of a component is *i*th, it is placed in the *i*th grid. For example, if the third component is placed on the display, it means that the component is placed on the third grid. *(compX[i] < compY[i]) && (grid[i].x < grid[i].y)* means that if the x-axis size of the component and grid is less than the y-axis size, the component is represented on the display as is. *(compX[i] > compY[i]] && (grid[i].y < grid[i].x)* means that if the x-axis size of the component and grid is greater than the y-axis size, the display is still represented on the display as is. *(compX[i] < compY[i]) && (grid[i].y < grid[i].x)* means that if the x-axis size of the component is less than the y-axis size and the x-axis size of the grid is greater than the y-axis size, the component’s x-axis and y-axis are swapped together to be represented on the display. *(compX[i] < compY[i]) && (grid[i].y < grid[i].x)* means that if the x-axis size of the component is greater than the y-axis size and the x-axis size of the grid is less than the y-axis size, the component’s x-axis and y-axis are swapped together to be represented on the display.

## 4. Performance Analysis 

In this paper, four experiments were conducted to validate the AVS. The first experiment was conducted in the vehicle and in the cloud with the HMM model to validate learning efficiency in the NVIDIA px2 driver environment. The second experiment was conducted to compare the learning time of HMM, RNN, and long short-term memory (LSTM). The third experiment was conducted to compare the computational time of Tacotron2, Deep Voice, and Deep Mind. The fourth experiment was conducted to compare the time taken for the vehicle’s instrument counter and DVM to visualize the vehicle’s information in real time. The experiment was conducted on a PC with Intel i5-7400 CPU, GTX1050 GPU, 8GB RAM, and Windows 10 Education OS.

### 4.1. Performance Analysis of HMM Learning

**Figure 10:**
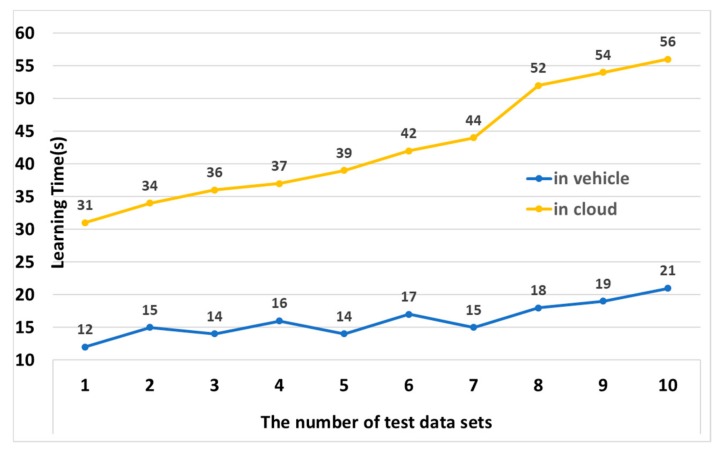
Performance analysis of HMM learning.

Figure 10 shows the time it took for the STS to learn the HMM in the cloud and in a vehicle. In this experiment, the time to learn the HMM was measured with 1 to 10 test datasets. In the vehicle, the number of test datasets did not have a significant impact on the learning time, but the learning time in the cloud was increased as the number of test sets increased. The trend line of learning time from the vehicle is 0.78182 and that of from the cloud is 2.84242. In the vehicle, the more learning data the HMM have, the more effective the HMM learning is. In addition, since the average learning time in a vehicle is about 2.5 times faster than in the cloud, this paper proposes using the HMM in the vehicle rather than in the cloud.

### 4.2. Performance Analysis of STS

**Figure 11:**
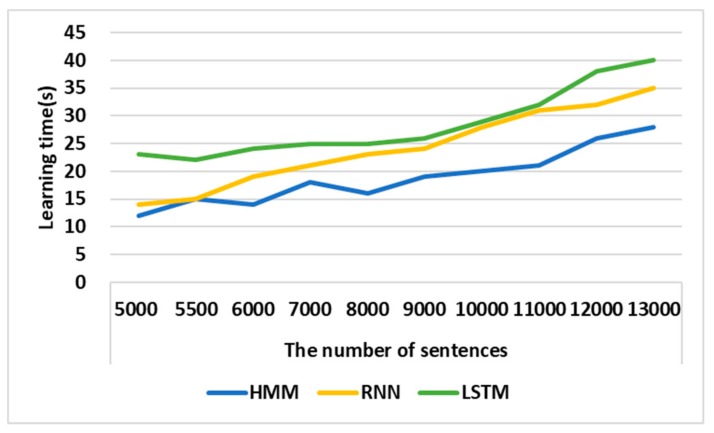
Performance Analysis of HMM. LSTM, long short-term memory.

Figure 11 shows the learning times of HMM, RNN, and LSTM. The learning time for each model was measured, increasing the sentences in the test dataset from 500 to 13,000. The sentences used in the experiment are included in the LJ training data sets. The experimental results show that the HMM was able to recognize speech faster than RNN and LSTM by 25% and by 42.86%, respectively. When the number of the test sentences was small, there was little difference in the learning time between the RNN and the HMM, but as the number of the test sentences grew larger, the time of the HMM learning become faster. Therefore, the HMM is the most appropriate to ensure the real-time of AVS speech recognition and voice transmission.

**Figure 12:**
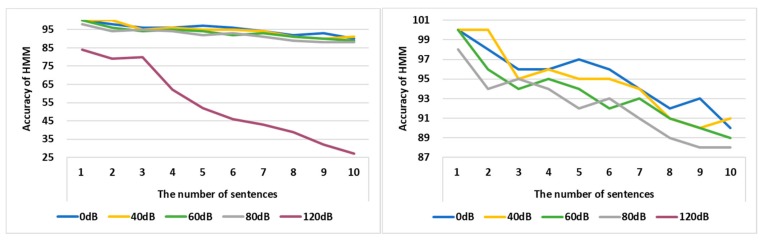
The accuracy of the HMM according to noise.

Figure 12 shows the accuracy of HMM when the sentences with noise were entered into the HMM. The sentences used in the experiment were included in the LJ training data sets and were not used in the HMM’s training. The accuracy is computed by comparing the difference between when a sentence is entered into the HMM and when it is output. The experiment was conducted using 1 to 10 sentences, and the sentences with the noise of 0 dB, 40 dB, 60 dB, 80 dB, and 120 dB were entered into the HMM.

In the left experiment of Figure 12, because the accuracy was significantly reduced in case of 120 dB, the 120 dB was excluded in the right experiment. The average accuracy of the HMM was 95% when sentences without noise were input into the HMM. That of the HMM was 95% when sentences with 40 dB noise were input into the HMM. The average accuracy of the HMM was 93% when sentences with 60 dB noise were input into the HMM. The average accuracy of the HMM was 92% when sentences with 80 dB noise were input into the HMM. The average accuracy of the HMM was 54% when sentences with 120 dB noise were input into the HMM. The average noise is between 60 and 70 dB when the vehicle is travelling at a speed of 60 to 80 km/h. The accuracy of the HMM can be above 90% even when 80 dB noise is mixed in a sentence, so the HMM can recognize the speech well even if noise occurs in the vehicle. 

### 4.3. Performance Analysis of TWS

**Figure 13:**
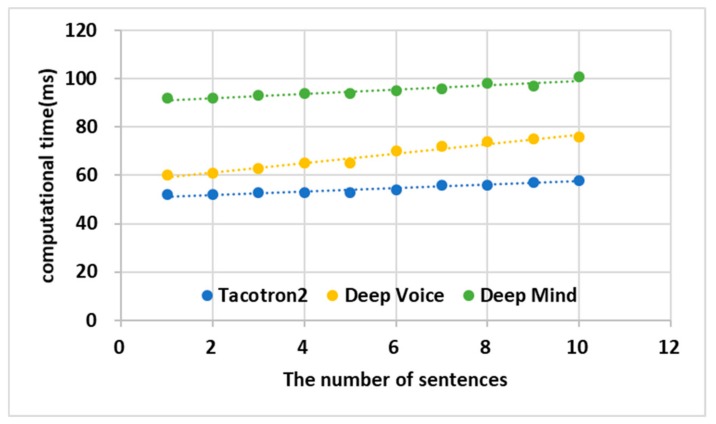
Performance analysis of HMM.

Figure 13 shows the computational time it took for the TTS engines Tacotron2, Deep Voice, and Deep Mind to convert text into voice when sentences were increased from 1 to 10. The sentences used in this experiment are those not used for learning among LJ Training data sets. Tacotron2 converted text into voice about 20 ms faster than Deep Voice and about 50 ms faster than Deep Mind. The TWS converted the vehicle’s data into voice using Tacotron2, so real time could be guaranteed. The computational time of the Deep Mind is about 1.5 times slower than the Deep Voice and Tacotron2. As test data sets get more, the difference of computational time between the Tacotron2 and Deep Voice gets bigger. The trend line of the Tacotron2 is 0.69091 and that of the Deep Voice line is 1.95758. Therefore, the AVS should use Tacotron2 to ensure real-time voice transmission.

### 4.4. Performance Analysis of DVM

**Figure 14:**
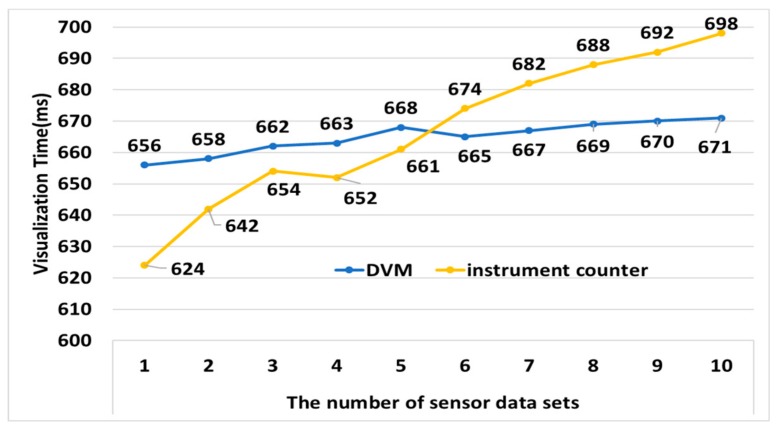
Performance analysis of DVM.

Figure 14 compares the time it took for the sensor data to be displayed on the vehicle’s instrument counter to analyze the performance of the DVM. Performance analysis used 1 to 10 datasets for visualization. The average time for the DVM to visualize sensor data was about 665 ms and for the instrument counter was about 667 ms. The DVM was about 2 ms faster than an existing instrument counter; it can visualize information without compromising the real time of an existing instrument counter. However, the instrument counter cannot display information more accurately than the DVM because it should display only the information that is visible in driving, and as test data sets gets more, it takes more time for the counter to visualize sensor data than DVM. When the number of sensor data sets to be visualized was 10, the DVM was about 20 ms faster than the instrument counter in visualizing sensor data. 

As a result, the AVS not only visualizes data intuitively without compromising the real time of an existing instrument, but also enables the vehicle data to be heard in real time for the blind. Therefore, the AVS will better prevent accidents than existing systems and help blind people check the condition of the vehicle. 

## 5. Conclusions

When blind and deaf people use fully autonomous vehicles as passengers, an intuitive and accurate visualization screen for the deaf should be provided, and an audifying system with speech-to-text (STT) and text-to-speech (TTS) functions should be provided for the blind. This paper proposes an audification and visualization system (AVS) for blind and deaf people that can store the graphic data collected from vehicle cameras, etc., and the in-vehicle sensor data and fault self-diagnosis data in the vehicle and select the desired data by using a touch interface and a speech recognizer. The AVS consists of three modules. The DCMM stores and manages the data collected from the vehicle. The ACM has a speech-to-text submodule (STS) that recognizes a user’s speech and converts it to text data, and a text-to-wave submodule (TWS) that converts the text data to speech. The DVM receives sensor data, self-diagnosis information, and a graphics library from the DCMM for visualization through the touch interface. The DVM provides an adaptive position of visualized data to fit the display on the vehicle. 

We conducted four experiments to validate the AVS. The first experiment was conducted in the vehicle and in the cloud with the HMM model to validate learning efficiency. The second experiment was conducted to compare the learning times of HMM, RNN, and LSTM. The third experiment was conducted to compare the computational time of Tacotron, Deep Voice, and Deep Mind. The fourth experiment was conducted to compare the time it took for the vehicle’s instrument counter and DVM to visualize the vehicle’s information in real time. According to the experimental results, the HMM was about 2.5 times faster than the cloud when it was learned in the NVIDIA px2 driver, the HMM used in STS was learned about 25% faster than the RNN and about 42% faster than the LSTM, and Tacotron2 used in TWS converted text to voice 20 ms faster than Deep Voice and 50 ms faster than Deep Mind. Finally, the DVM visualized the data 2 ms faster than existing instrument clusters. Therefore, the AVS can express more information without compromising the real time of existing systems. 

However, the AVS was not tested on actual vehicles and mean opinion score (MOS) measurements, which are voice quality criteria, were not done when data was converted to voice. If the AVS is applied to real vehicles, it will not only make autonomous vehicles more readily available to blind and deaf people, but will also increase the stability of existing autonomous vehicles. In addition, vehicles without displays were not considered because the AVS would generate a GUI based on the displays inside the vehicle. The future AVS should objectively evaluate the quality of data converted into voice by MOS measurements and be applied to actual vehicles, and how the system will be applied to devices other than smartphones or in-vehicle displays should be studied.

## Figures and Tables

**Table 1:** Components of HMM.

Set Name	Set Contents	Meaning
Q	{q_1_, q_2_, …, q_N_}	Set of hidden states
Y	{y_1_, y_2_, …, y_M_}	Set of observed values in a hidden state
π	{ π_1_, π_2_, …, π_N_|R^N^}	Set of initial probabilities p(q_i_) with the probability of initial state q_i_
T	{T_12_, T_21_, …., T_NM_, T_MN_|R^NxN^}	Set of transition probabilities p (q_j_|q_i_) indicating the probability of moving from q_i_ to q_j_
E	{E_11_, E_12_, …, E_NM_|R^NxM^}	Set of assignment probabilities p(y_j_|q_i_) indicating the probability that y_j_ will occur in q_i_
θ	{π, T, E}	HMM parameter

**Table 2:** Observed values that can be output from the speech-to-text submodule (STS).

Observed Value	Data Name	Observed Value	Data Name
y_1_	Speed	y_7_	Driving distance
y_2_	RPM	y_8_	Timing belt
y_3_	Tire	y_9_	Spark plug
y_4_	Steering wheel	y_10_	Air conditioner
y_5_	Engine oil	y_11_	Brake pad
y_6_	Coolant		
